# Preoperative anxiety and its impact on surgical outcomes: A systematic review and meta-analysis

**DOI:** 10.1017/cts.2025.6

**Published:** 2025-01-17

**Authors:** Mohamed A. Shebl, Eman Toraih, Menna Shebl, Ahmed Mosaad Tolba, Parisa Ahmed, Harshdeep Singh Banga, Mohab Orz, Mahmoud Tammam, Keroles Saadalla, Mohamed Elsayed, Mennatallah Kamal, Mohamed Abdulla, Ahmed Ibrahim Eldessouky, Yousef Tarek Moustafa, Omar Ahmed Mohamed, Hani Aiash

**Affiliations:** 1 Faculty of Medicine, Cairo University, Kasr Al-Ainy, Cairo, Egypt; 2 Tulane University School of Medicine, New Orleans, LA, USA; 3 Faculty of Medicine, Suez Canal University, Ismailia, Egypt; 4 Upstate Medical University, Syracuse, USA; 5 Faculty of Medicine, Modern University for Technology and Information, Cairo, Egypt; 6 Faculty of Medicine, Alexandria University, Alexandria, Egypt; 7 Virginia Commonwealth University, Richmond, USA; 8 Dnipropetrovsk Medical Institute of Conventional and Alternative Medicine, Dnipropetrovsk, Ukraine

**Keywords:** Preoperative anxiety, delirium, STAI, mYPAS, propofol

## Abstract

**Background:**

Preoperative anxiety is a common phenomenon affecting 60–80% of surgical patients, with potential implications for surgical outcomes. Despite its prevalence, there remains a lack of consensus on its precise effects and optimal management strategies.

**Objective:**

This meta-analysis aimed to synthesize current evidence on the impact of preoperative anxiety on various surgical outcomes, including anesthetic and analgesic requirements, delirium, recovery times, and pain.

**Methods:**

We conducted a comprehensive literature search and meta-analysis of studies examining the relationship between preoperative anxiety and surgical outcomes. Standardized mean differences (SMD), correlation (COR), and odds ratios (OR) with 95% confidence intervals were calculated.

**Results:**

Our analysis revealed significant associations between preoperative anxiety and increased anesthetic requirements (SMD = 0.67, 95% CI: 0.32–1.01) and analgesic requirements (SMD = 0.89, 95% CI: 0.65–1.12). Preoperative anxiety was associated with postoperative delirium in adults (OR = 1.90, 95% CI: 1.11–3.26), unlike the pediatric population. Preoperative anxiety was associated with prolonged time to reach Modified Aldrete Score of 9 (SMD = 0.79, 95% CI: 0.50–1.07) and extubation time (SMD = 0.89, 95% CI: 0.58–1.21). Preoperative anxiety was positively correlated with propofol consumption (STAI-S COR = 0.35, 95%CI: 0.15–0.55). No significant association between preoperative anxiety and postoperative pain was found.

**Conclusions:**

This meta-analysis provides evidence for the wide-ranging effects of preoperative anxiety on surgical outcomes. The findings emphasize the need for routine preoperative anxiety screening and the development of targeted interventions. Future research should focus on long-term impacts and the effectiveness of various anxiety management strategies.

## Introduction

Surgical procedures, while often necessary for health improvement, can be a significant source of stress and anxiety for patients. Preoperative anxiety, characterized by feelings of tension, apprehension, nervousness, and worry in the period leading up to surgery, is a common phenomenon affecting an estimated 60–80% of surgical patients [1]. This psychological state can have far-reaching implications, potentially influencing not only the patient’s immediate well-being but also their surgical outcomes and recovery trajectory [2–4].

The impact of preoperative anxiety on surgical outcomes has been a subject of growing interest in the medical community over the past few decades. Several studies have suggested that heightened anxiety levels before surgery may be associated with a range of adverse outcomes, including increased pain perception, higher anesthetic requirements, and prolonged recovery times [5]. Moreover, there is emerging evidence linking preoperative anxiety to more severe complications such as postoperative delirium and cardiac events [6].

However, the relationship between preoperative anxiety and surgical outcomes is complex and multifaceted. The manifestation and impact of anxiety may vary depending on factors such as the type of surgery, patient demographics, and the specific outcomes being measured [7,8]. Furthermore, the tools used to assess preoperative anxiety are diverse, ranging from general anxiety scales to surgery-specific measures, which can lead to variability in findings across studies [9].

Despite the potential significance of preoperative anxiety, there remains a lack of consensus on its precise effects and the best strategies for its management. While some healthcare systems have implemented routine preoperative anxiety screening and interventions, others have yet to incorporate such practices into standard care protocols. This variability in practice underscores the need for a comprehensive understanding of the relationship between preoperative anxiety and surgical outcomes.

To address this gap in knowledge, we conducted a systematic review and meta-analysis of the available literature on preoperative anxiety and its association with various surgical outcomes. Our study aimed to synthesize current evidence on the impact of preoperative anxiety on anesthetic and analgesic requirements, the relationship between preoperative anxiety and postoperative delirium, the effectiveness of different anxiety scales in predicting surgical outcomes, and the influence of preoperative anxiety on recovery times.

By consolidating and analyzing data from multiple studies, we sought to provide a more comprehensive understanding of how preoperative anxiety influences surgical outcomes. This knowledge is crucial for informing evidence-based practices in preoperative care, potentially leading to improved patient experiences, optimized resource utilization, and enhanced surgical outcomes.

## Methods

This systematic review and meta-analysis were performed and reported following the Cochrane Collaboration Handbook for Systematic Reviews of Interventions and the Preferred Reporting Items for Systematic Reviews and Meta-Analysis Statement guidelines.

### Search strategy

We conducted a comprehensive literature search in PubMed, Scopus, and Web of Science from inception to 30th March 2024. The search strategy employed key terms such as “preoperative anxiety” and “delirium” to identify relevant studies. The complete search strategy is provided in Supplementary Information Methods S3. After removing duplicates, two authors (A.T. and P.A.) independently screened titles and abstracts and assessed full-text articles for inclusion based on predefined criteria. Discrepancies were resolved through discussion with the senior author. Additionally, we reviewed reference lists of included studies to identify any further eligible articles.

### Eligibility criteria

Studies were included if they met the following criteria: (1) published in English; (2) reported data on effect of preoperative anxiety on perioperative outcomes; and (3) employed observational study designs including cross-sectional, case-control, and cohort studies (both prospective and retrospective). We excluded editorials, letters to the editor, commentaries, reviews, systematic reviews, meta-analyses, case reports, case series, and animal studies.

### Data extraction

Two authors independently extracted data from the included studies using a standardized form. Extracted information included the first author’s surname, publication year, study design, sample characteristics (age, gender, and race), and baseline comorbidities. Outcomes of interest included the effect of anxiety on anesthetic and analgesic drug requirements, delirium incidence, recovery times, and postoperative pain.

### Quality assessment

For included observational studies, the Newcastle–Ottawa scale (NOS) was used to assess the quality of each study included [10]. This scale evaluates studies based on selection, comparability, and outcome/exposure, with a maximum score of 9 points. Studies were categorized as low risk of bias (score ≥ 7), intermediate risk (score 4–6), or high risk (score < 4).

### Statistical analysis

Continuous variables were reported as mean ± standard deviation (SD), and binary variables were reported as totals and percentages. For meta-analysis, we used a Mantel-Haenszel random-effects model for binary outcomes, calculating odds ratio (OR) and 95% confidence interval (CI), while mean differences (MD) were computed for continuous variables. The standardized mean difference (SMD) was employed to measure the effect size across studies that reported different surgeries or measuring systems. The DerSimonian and Laird (DL) method estimated between-study variance (τ^2^). For correlation analyses, we calculated correlation coefficients (COR) with 95% confidence intervals. A two-tailed *p*-value of < 0.05 was considered statistically significant.

Heterogeneity was assessed using Cochrane’s Q test and I^2^ statistics, with *p* ≤ 0.05 indicating statistical significance. The consistency of the studies was determined based on I^2^ values of 0%, ≤ 25%, ≤ 50%, and > 50%, indicating no observed, low, moderate, and substantial heterogeneity, respectively. Subgroup analyses were conducted to examine the effects of preoperative anxiety in different populations (pediatric vs. adult) and for different outcomes (e.g., postoperative pain at various time points). We performed sensitivity analysis by leave-one-out analysis according to specified criteria in the supplementary file. All analyses were conducted using Revman version 5.4 and R version 4.4.1 with “*meta*” and “*dmetar*” for all calculations and graphics [11–13]. For correlation analyses, we used the “metacor” function from the “meta” package in R to perform meta-analysis of correlation coefficients.

## Results

### Characteristics of eligible studies

**Figure 1.**
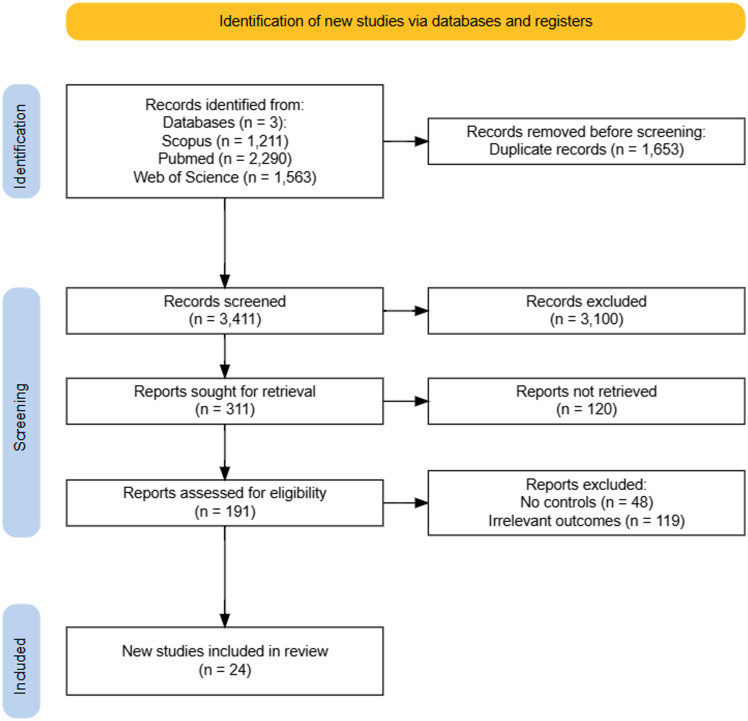
Preferred Reporting Items for Systematic Reviews and Meta-Analysis flow chart of study screening and inclusion.

Our initial search yielded 5064 articles (Figure 1). After removing duplicates and screening titles and abstracts, we retrieved 191 articles for full-text review. Ultimately, 24 articles [14–37] met the inclusion criteria and were included in the analysis. The studies included 3,388 patients. Table 1 provides a detailed summary of the baseline characteristics of the included studies.

**Table 1.** Baseline

Study	Surgery	Sample Size	Age (Mean ± SD)	Male n(%)	Anxiety Group, n(%)
Ersoy 2024	Spinal surgery	80	–	38 (47.5%)	–
Sahin 2023	Oocyte retrieval	131	34.15 ± 5.39	0 (0%)	60
Prayunato 2023	Elective surgery	100	6.6 ± 3.4	59 (59%)	–
Mou 2023	lumbar disc herniation	863	68.2 ± 8.3	604 (70%)	225
Arunalatha 2023	Elective surgery	100	45.31 ± 2.34	52 (52%)	50
Kashif 2022	Cardiac surgery	100	58.24 ± 10.03	68 (68%)	64
Fukunaga 2022	cardiovascular surgery	168	74.9 ± 6.1	93 (55.4%)	–
Ali 2022	Multiple infra-umbilical surgeries	250	–	230 (92%)	–
Ren 2021	Elective Orthopedic Surgery	263	74.2 ± 7.3	74 (28.1%)	40 (15.2%)
Ma 2021	Hip arthroplasty	325	52.1 ± 13.8	60 (63.2%)	95
Inal 2021	Endoscopic Ultrasound	80	53.79 ± 9.99	37 (46.3%)	–
Cheng 2021	Cardiac Surgery	152	63.07 ± 11.17	104 (68.42%)	–
Uysal 2020	laparoscopic cholecystectomy	79	40.34 ± 14.40	43 (54.4%)	42
Jooma 2020	Dental surgery	91	3.9 ± 0.9	58 (63.7%)	63
Milisen 2020	Cardiac surgery	190	75.7 ± 5.9	99 (52.1%)	59 (31.1%)
Wada 2019	tumor resections	91	66.0 ± 10.0	62 (68.1%)	14
Masri 2018	Multiple surgeries	52	–	23 (44.2%)	–
Barreto 2018	Multiple surgeries	100	–	66 (66%)	41 (41%)
Manjunatha 2017	elective surgery under general anesthesia	42	–	13 (30.9%)	–
Van Grootven B 2016	Traumatic hip fracture surgery	86	80.1 ± 6.8	21 (24.4%)	–
Ali 2014	laparoscopic cholecystectomy	80	46.42 ± 10.96	33 (41.25%)	31
Gras 2010	Gynecological surgery	45	–	–	–
Osborn 2004	Dental surgery	25	–	–	9
Maranets 1999	bilateral laparoscopic tubal ligation	57	30.74 ± 5.19	–	–

### Quality assessment

Individual appraisal of included studies is reported in Supplementary Table S4. Seven of studies were given a score of 4 to 6. Furthermore, 17 studies had scores of ≥ 7 and were deemed as high-quality studies.

### Anesthesia-related outcomes

**Figure 2.**
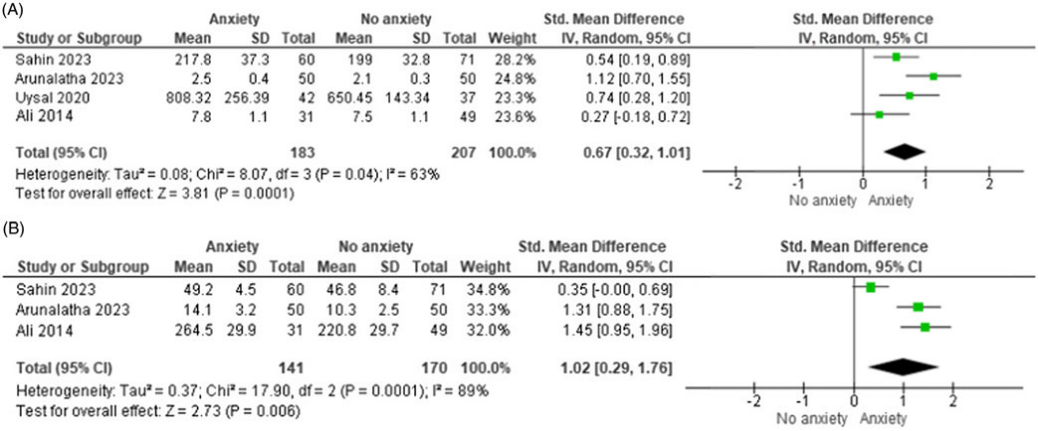
Anesthesia-related outcomes. (A) Forest plot of anesthetic drug dose requirements. The *x*-axis represents the standardized mean difference (SMD) in anesthetic drug dose between anxious and non-anxious patients. (B) Forest plot of analgesic drug dose requirements. The *x*-axis represents the SMD in analgesic drug dose between anxious and non-anxious patients. For both plots, squares represent individual studies, with size proportional to study weight. Diamond represents the pooled effect size. Horizontal lines represent 95% confidence intervals.

Our pooled analysis demonstrated a significant increase in anesthetic drug requirements among individuals with per-operative anxiety (SMD = 0.67, 95%CI: 0.32–1.01) with moderate heterogeneity (I^2^ = 63%) (Figure 2A). Similarly, we observed a significant increase in analgesic drug requirements among individuals with preoperative anxiety (SMD = 1.02, 95%CI: 0.29–1.76), with significant heterogeneity (I^2^ = 89%) (Figure 2B).

### Postoperative delirium

**Figure 3.**
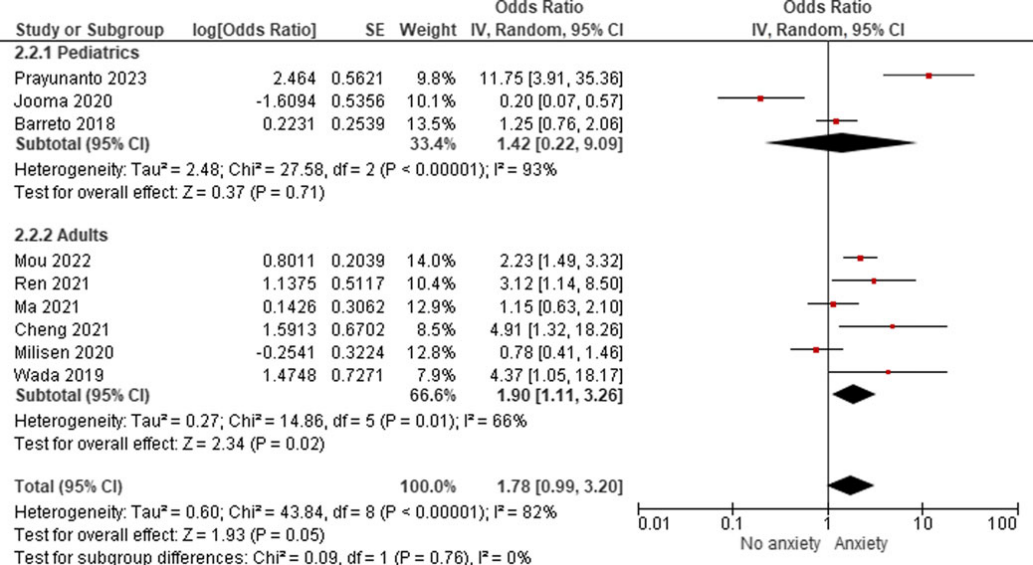
Postoperative delirium and anxiety scales. Forest plot of postoperative delirium occurrence. The *x*-axis represents the odds ratio of delirium occurrence in anxious versus non-anxious patients. Squares represent individual studies, with size proportional to study weight. Diamond represents the pooled effect size. Horizontal lines represent 95% confidence intervals.

Our pooled analysis of studies classifying individuals dichotomously revealed increased odds of delirium occurrence among those classified as anxious preoperatively (OR = 1.89, 95%CI: 1.00–3.55) with significant heterogeneity (I^2^ = 80%) (Figures 3). Subgroup analysis showed varying results across different populations. In the pediatric population, our analysis revealed a non-significant association between anxiety status and emergence delirium incidence (OR = 1.42, 95% CI: 0.22–9.09). Conversely, in the adult population, we found a significant increase in the odds of postoperative delirium among individuals with preoperative anxiety (OR = 2.03, 95% CI: 1.49–2.76).

### Anxiety scales

Our pooled analysis of anxiety scales revealed varying associations between anxiety scores and odds of delirium across different measures (Table 2). The Modified Yale Preoperative Anxiety Scale (mYPAS) showed a significant positive association (OR = 1.23, 95% CI: 1.16–1.30). However, other scales demonstrated non-significant associations: State-Trait Anxiety Inventory - State (STAI-S) (OR = 0.98, 95% CI: 0.89–1.08), State-Trait Anxiety Inventory - Trait (STAI-T) (OR = 1.01, 95% CI: 0.93–1.11), Amsterdam Preoperative Anxiety and Information Scale - Anxiety (APAIS-A) (OR = 0.97, 95% CI: 0.88–1.07), Hospital anxiety and depression scale - Anxiety (HADS-A) (OR = 1.10 95% CI: 1.01–1.21) and State-Trait Anxiety Inventory-6 (STAI-6) (OR = 1.18, 95% CI: 0.89–1.56). Thus, most scales showed non-significant associations, except for mYPAS and HADS-A, which demonstrated a significant positive association with delirium odds.

**Table 2.** Anxiety scales and odds of delirium

Scale	OR	CI (95%)	Reference
STAI-S	0.98	0.89-1.08	[20]
STAI-T	1.01	0.93-1.11	[20]
mYPAS	1.23	1.16-1.30	[21]
APAIS-A	0.97	0.88-1.07	[28]
STAI-6	1.18	0.89-1.56	[33]
HADS-A	1.10	1.01-1.21	[23]

Abbreviations: STAI = State-Trait Anxiety Inventory; STAI-S = STAI-state subsystem; STAI-T = STAI-trait subsystem; STAI-6 = State-Trait Anxiety Inventory - six items (shortened state subsystem); BAI = Beck’s Anxiety Inventory; mYPAS = modified Yale Pediatric Anxiety Scale; MAS = Modified Aldrete Score; APIAS-A = Amsterdam Preoperative Anxiety and Information Scale – Anxiety; HADS-A = Hospital Anxiety and Depression Scale - Anxiety subsystem.

### Recovery times

**Figure 4.**
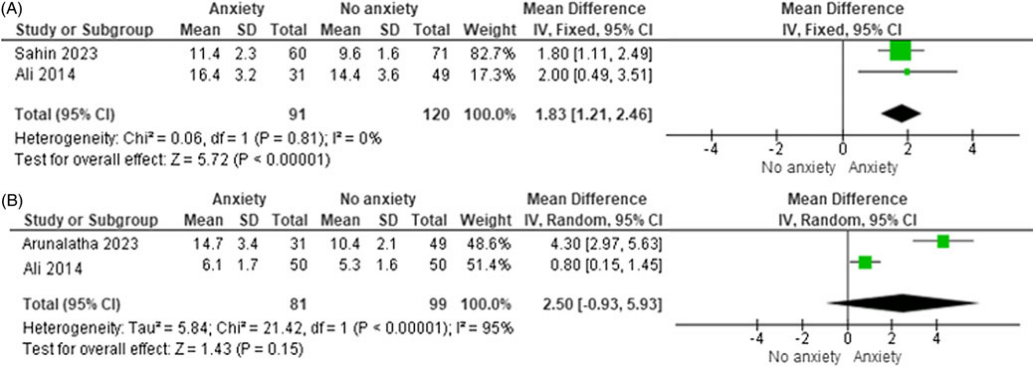
Recovery times. (A) Forest plot of time to reach Modified Aldrete Score of 9. (B) Forest plot of extubation time. The *x*-axis represents the mean difference in minutes between anxious and non-anxious patients. For both plots, squares represent individual studies, with size proportional to study weight. Diamond represents the pooled effect size. Horizontal lines represent 95% confidence intervals.

We found a significant increase in the time to reach a MAS score of 9 among individuals with preoperative anxiety (MD = 1.83 minutes, 95%CI:1.21–2.46) (Figure 4A). Our analysis demonstrated a significant increase in extubation time among individuals with preoperative anxiety (MD = 2.50 minutes, 95%CI:-0.93–5.93). This analysis showed significant heterogeneity (I^2^ = 95%) (Figure 4B).

### Propofol consumption

**Figure 5.**
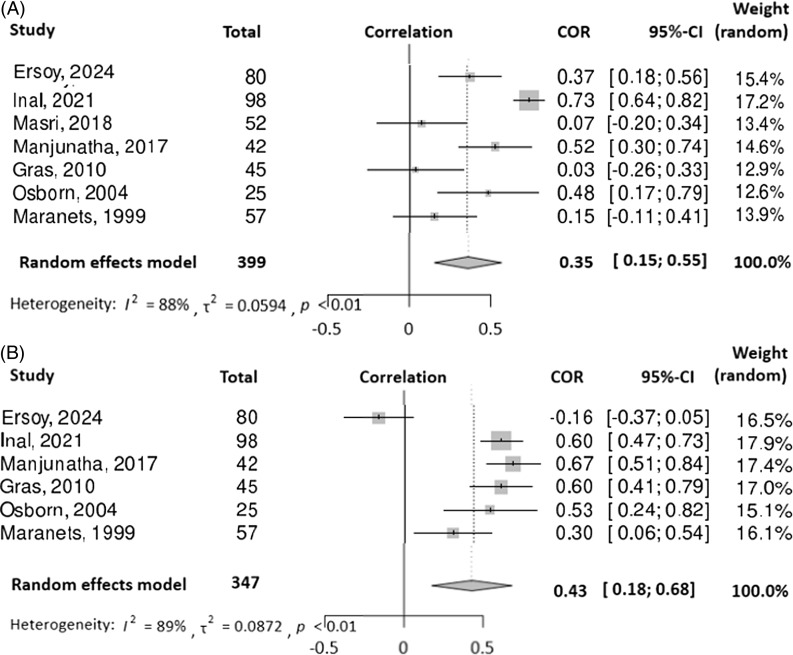
(A) Forest plot and meta-analysis of correlation coefficients testing the relationship between pre-op state anxiety (STAI-S scale) and propofol consumption (B) forest plot and meta-analysis of correlation coefficients testing the relationship between preop trait anxiety (STAI-T scale) and propofol consumption. For both plots, squares represent individual studies, with size proportional to study weight. Diamond represents the pooled effect size. Horizontal lines represent 95% confidence intervals.

Our pooled analysis demonstrated a significant correlation between STAI-S score and propofol consumption (COR = 0.35, 95%CI: 0.15–0.55) with significant heterogeneity (I^2^ = 88%) (Figure 5A). Similarly, we observed a significant correlation between STAI-T score and propofol (COR = 0.43, 95%CI: 0.18–0.68), with significant heterogeneity (I^2^ = 89%) (Figure 5B).

### Pain

**Figure 6.**
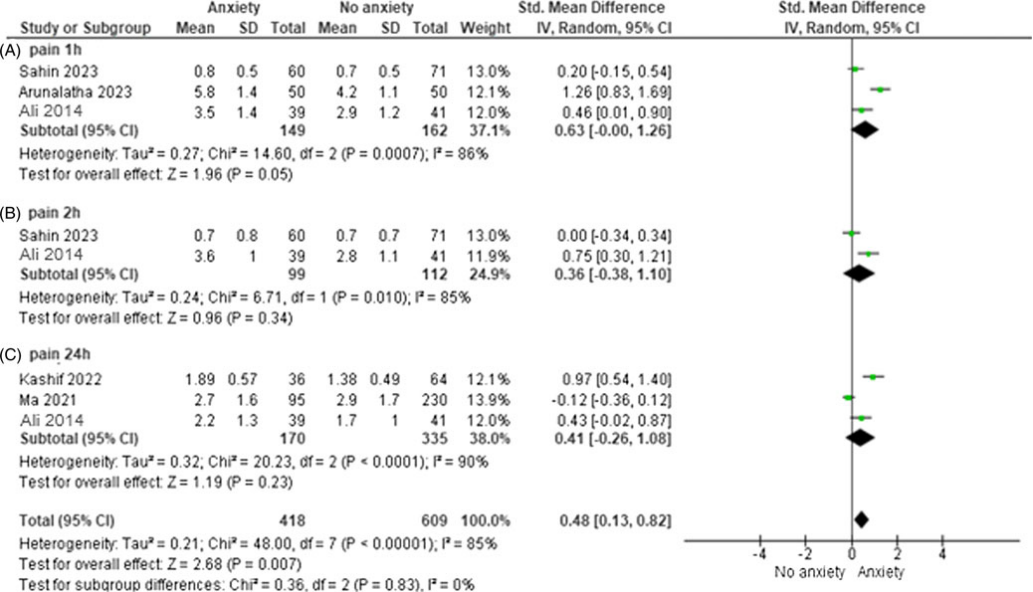
Pain (A) forest plot of pain 1h postoperatively (B) forest plot of pain 2h postoperatively (C) forest plot of pain 24h postoperatively. For all plots, squares represent individual studies, with size proportional to study weight. Diamond represents the pooled effect size. Horizontal lines represent 95% confidence intervals.

Our pooled analysis demonstrated a non-significant increase in postoperative pain 1h among individuals with per-operative anxiety (SMD = 0.63, 95%CI: 0–1.26). This analysis showed moderate heterogeneity (I^2^ = 86%) (Figure 6A). Similarly, we observed non-significant increase in postoperative pain 2h among individuals with preoperative anxiety (SMD = 0.36, 95%CI: −0.38–1.10), with significant heterogeneity (I^2^ = 85%) (Figure 6B). Our analysis revealed non-significant increase in postoperative pain 24h among individuals with preoperative anxiety (SMD = 0.36, 95%CI: −0.38–1.10), with significant heterogeneity (I^2^ = 90%) (Figure 6C).

### Sensitivity analysis

Leave-one-out analysis was performed using the criteria explained in the supplementary Figure 5.

## Discussion

Our meta-analysis provides compelling evidence for the significant impact of preoperative anxiety on various surgical outcomes. These findings underscore the critical importance of recognizing and addressing anxiety in surgical patients, as it demonstrably influences anesthetic requirements, postoperative delirium risk, and recovery times. The observed increase in anesthetic and analgesic drug requirements among anxious patients aligns with previous research suggesting that anxiety can alter pain perception and drug metabolism [38–41].

The increased need for anesthesia and the consumption of propofol observed in our meta-analysis are critical findings that warrant further discussion. Sensitivity analyses by removing the only study with minor procedure [15] further increased the magnitude of association. Increased dosages of anesthetics and analgesics, such as propofol, can lead to several specific adverse effects. For instance, higher doses of propofol can increase the risk of hypotension, respiratory depression, and prolonged recovery times due to its sedative properties [42–47]. Additionally, frequent administration of higher doses can increase the likelihood of developing propofol infusion syndrome, a rare but potentially fatal condition characterized by metabolic acidosis, rhabdomyolysis, and cardiovascular collapse [48–51].

From an economic perspective, the increased consumption of anesthetics also has significant implications for healthcare costs. The precise cost implications can vary widely depending on the healthcare setting and country. However, rough estimates suggest that increased drug usage could result in substantial additional costs for hospitals. For instance, studies indicate that higher anesthesia requirements can lead to increased expenditure on drug procurement, extended operating room times, and longer hospital stays due to slower recovery [52,53]. Specifically, propofol, while cost-effective per dose, can accumulate significant costs when used in higher quantities over multiple surgeries [54–56].

Our analysis of postoperative delirium reveals a nuanced relationship with preoperative anxiety. The significant association found in the overall population, particularly in adults, corroborates earlier studies linking psychological distress to post-surgical cognitive complications [57,58]. The analysis revealed that association between preoperative anxiety and delirium in pediatrics was statistically insignificant. This is closely associated with the high heterogeneity in the result. However, sensitivity analysis removing the only study with minor surgery [27] didn’t change the non-significance of the association another explanation could be the small sample size and relatively low number of studies in this group. The non-significant finding in the pediatric population is intriguing and merits further investigation. Potential explanations may lie in children’s neuroplasticity or differences in anxiety manifestation, which could act as mediating factors [59]. The varying associations between different anxiety scales and delirium risk highlight the importance of tool selection in preoperative assessments. The significant association found with the Modified Yale Preoperative Anxiety Scale (mYPAS) suggests its potential utility for delirium risk stratification. Conversely, the non-significant findings with other scales underscore the complexity of anxiety measurement and emphasize the need for further validation of these tools in surgical contexts.

Our study highlights a significant association between preoperative anxiety and both extubation time and the time to reach a Modified Aldrete Score (MAS) of 9. These findings are consistent with previous research, which has demonstrated that higher levels of preoperative anxiety can negatively impact various aspects of postoperative recovery.

The correlation between preoperative anxiety and prolonged extubation time suggests that anxious patients may experience delayed recovery from anesthesia. This delay could be attributed to the physiological effects of anxiety, such as increased sympathetic nervous system activity, which may interfere with the smooth transition from anesthesia to wakefulness [60].

The Modified Aldrete Score is a widely used tool to assess recovery from anesthesia, with a score of 9 indicating readiness for discharge from the post-anesthesia care unit. Our results indicate that patients with higher preoperative anxiety take longer to achieve this score, suggesting a slower overall recovery process. This finding aligns with the study by [60], which found a significant correlation between preoperative anxiety and delayed recovery times, including the time to reach a MAS of 9 [60].

The implications of these findings are substantial. Delayed extubation and prolonged recovery times can increase the risk of postoperative complications, extend hospital stays, and elevate healthcare costs. Therefore, it is crucial to identify and manage preoperative anxiety effectively. Interventions such as preoperative counseling, anxiolytic medications, and relaxation techniques could be beneficial in mitigating the adverse effects of anxiety on postoperative recovery.

The findings of this study indicate that preoperative anxiety does not have a significant association with postoperative pain. This result contrasts with several previous studies that have suggested a link between preoperative anxiety and increased postoperative pain levels [6,61]. However, after conducting sensitivity analysis by removing the only study with minor procedure (Sahin et al. 2023 [15]), the association of pain 1h postoperatively and preoperative anxiety became significant.

One possible explanation for this discrepancy could be the differences in the methodologies used across studies. For instance, variations in the scales used to measure anxiety and pain, the timing of these measurements, and the types of surgeries performed might contribute to differing outcomes. In our study, multiple scales were used for anxiety assessment which might yield different results [6].

Another factor to consider is the role of perioperative care and pain management protocols. Advances in anesthesia and analgesia techniques, as well as enhanced recovery protocols, may mitigate the impact of preoperative anxiety on postoperative pain. Our study population received standardized pain management, which could have minimized the variability in pain outcomes related to anxiety levels [61].

The implications of our findings are significant for clinical practice. They suggest that while managing preoperative anxiety is important for overall patient well-being, it may not necessarily translate to reduced postoperative pain. Therefore, healthcare providers should continue to focus on comprehensive pain management strategies that address multiple aspects of patient care.

A comprehensive meta-analysis of perioperative anxiolytic medications highlights the efficacy and safety profiles of various drugs. **Benzodiazepines**, such as alprazolam, diazepam, lorazepam, midazolam, and triazolam, are frequently used due to their rapid onset and short duration of action. However, The association between postoperative delirium and benzodiazepine is a contentious topic, as study results vary, indicating a lack of consensus [62–64]. **Melatonin** has emerged as a promising alternative, demonstrating significant reductions in preoperative anxiety without substantial adverse effects [65].

The choice of anxiolytic medication is influenced by the type of anesthesia planned [66], as certain medications may interact with anesthetic agents. However, effective communication between the patient, surgeon, and anesthesiologist is crucial to ensure the safest and most effective anxiolytic regimen is chosen.

Future research should aim to further elucidate the relationship between preoperative anxiety and postoperative outcomes by exploring different patient populations, surgical procedures, and anxiety and pain assessment tools. Longitudinal studies that track patients over extended periods could provide deeper insights into how preoperative anxiety might affect long-term recovery and pain management.

## Limitations

Several limitations of our study should be acknowledged. The significant heterogeneity observed in some analyses suggests considerable variability across studies, which could be attributed to differences in patient populations, surgical procedures, or anxiety assessment methods. Additionally, the observational nature of the included studies limits our ability to establish causality.

One of the primary limitations of this study is the confounding effect of the type of surgery on the observed differences in postoperative delirium between pediatric and adult patients. The data for pediatric surgeries being derived from dental procedures introduces a significant confounding effect, as the effect of patient age is intertwined with the nature of minor versus major surgeries. However, we conducted sensitivity analysis as discussed earlier with change in results explained. Another limitation is the high heterogeneity in pediatric subgroup analysis. Although [27] were excluded for using a minor procedure, this didn’t changes the insignificance of association between pediatric population anxiety and risk of postoperative delirium. This insignificance might be due to the small sample size. More studies need to be conducted on the pediatric population to explore the effect of anxiety on delirium.

## Conclusion

In conclusion, our meta-analysis provides robust evidence for the wide-ranging effects of preoperative anxiety on surgical outcomes. These findings emphasize the pressing need for routine preoperative anxiety screening and the development of targeted interventions to mitigate its impact. By addressing preoperative anxiety, healthcare providers may significantly improve patient experiences, optimize resource utilization, and ultimately enhance surgical outcomes. Future research addressing the identified priorities will further enhance our understanding of preoperative anxiety and lead to the development of more effective strategies to mitigate its impact on surgical outcomes.

## Supporting information

Shebl et al. supplementary material Shebl et al. supplementary material

## Data Availability

Data needed are available within the manuscript. Raw data will be shared upon optimum request.

## Supplementary material

The supplementary material for this article can be found at https://doi.org/10.1017/cts.2025.6.
