# Supplementary material for: Preoperative anxiety and its impact on surgical outcomes: A systematic review and meta-analysis
Source: J Clin Transl Sci. 2025 Jan 17;9(1):e33. doi: 10.1017/cts.2025.6 (PMC11883570; doi:10.1017/cts.2025.6)
Supplement: Shebl et al. supplementary material [file S2059866125000068sup001.docx]

## Supplementary Table S1.PRISMA 2020 Main Checklist.

| **Topic** | **No.** | **Item** | **Location where item is reported** |
| --- | --- | --- | --- |
| **TITLE** | | | |
| **Title** | 1 | Identify the report as a systematic review. | pg. 1 in the MS |
| **ABSTRACT** | | | |
| **Abstract** | 2 | See the PRISMA 2020 for Abstracts checklist |  |
| **INTRODUCTION** | | | |
| **Rationale** | 3 | Describe the rationale for the review in the context of existing knowledge. | pg. 6 in the MS |
| **Objectives** | 4 | Provide an explicit statement of the objective(s) or question(s) the review addresses. | pg. 6,7 in the MS |
| **METHODS** | | | |
| **Eligibility criteria** | 5 | Specify the inclusion and exclusion criteria for the review and how studies were grouped for the syntheses. | pg. 8 in the MS |
| **Information sources** | 6 | Specify all databases, registers, websites, organisations, reference lists and other sources searched or consulted to identify studies. Specify the date when each source was last searched or consulted. | pg. 7,8 in the MS |
| **Search strategy** | 7 | Present the full search strategies for all databases, registers and websites, including any filters and limits used. | pg. 7,8 in MS, pg 6 in the suppl. |
| **Selection process** | 8 | Specify the methods used to decide whether a study met the inclusion criteria of the review, including how many reviewers screened each record and each report retrieved, whether they worked independently, and if applicable, details of automation tools used in the process. | pg. 8 in the MS |
|  |  |  |  |
|  |  |  |  |
| **Data collection process** | 9 | Specify the methods used to collect data from reports, including how many reviewers collected data from each report, whether they worked independently, any processes for obtaining or confirming data from study investigators, and if applicable, details of automation tools used in the process. | pg. 7 in the MS. |
|  |  |  |  |
| **Topic** | **No.** | **Item** | **Location where item is reported** |
| **Data items** | 10a | List and define all outcomes for which data were sought. Specify whether all results that were compatible with each outcome domain in each study were sought (e.g. for all measures, time points, analyses), and if not, the methods used to decide which  results to collect. | pg. 8 in the MS |
|  | 10b | List and define all other variables for which data were sought (e.g. participant and intervention characteristics, funding sources). Describe any assumptions made about any missing or unclear information. | Pg. 8 in the MS, |
| **Study risk of bias assessment** | 11 | Specify the methods used to assess risk of bias in the included studies, including details of the tool(s) used, how many reviewers assessed each study and whether they worked independently, and if applicable, details of automation tools used in the process. | pg. 8,9 in the MS |
| **Effect measures** | 12 | Specify for each outcome the effect measure(s) (e.g. risk ratio, mean difference) used in the synthesis or presentation of results. | pg. 9 in the MS |
| **Synthesis methods** | 13a | Describe the processes used to decide which studies were eligible for each synthesis (e.g. tabulating the study intervention characteristics and comparing against the planned groups for each synthesis (item 5)). | N/A |
|  | 13b | Describe any methods required to prepare the data for presentation or synthesis, such as handling of missing summary statistics, or data conversions. | pg. 9 in the MS |
|  | 13c | Describe any methods used to tabulate or visually display results of individual studies and syntheses. | Pg. 9 in the MS |
|  | 13d | Describe any methods used to synthesize results and provide a rationale for the choice(s). If meta-analysis was performed, describe the model(s), method(s) to identify the presence and extent of statistical heterogeneity, and software package(s) used. | pg.9 in the MS. |
|  | 13e | Describe any methods used to explore possible causes of heterogeneity among study results (e.g. subgroup analysis, meta- regression). | pg.9 in the MS; |
|  | 13f | Describe any sensitivity analyses conducted to assess robustness of the synthesized results. | pg. 9 in the MS. |
| **Reporting bias assessment** | 14 | Describe any methods used to assess risk of bias due to missing results in a synthesis (arising from reporting biases). | pg. 8,9 in the MS; |
| **Certainty assessment** | 15 | Describe any methods used to assess certainty (or confidence) in the body of evidence for an outcome. | N/A |

| **Topic** | **No.** | **Item** | **Location where item is reported** |
| --- | --- | --- | --- |
| **RESULTS** |  |  | |
| **Study selection** | 16a | Describe the results of the search and selection process, from the number of records identified in the search to the number of studies included in the review, ideally using a flow diagram. | Fig. 1 |
|  | 16b | Cite studies that might appear to meet the inclusion criteria, but which were excluded, and explain why they were excluded. | N/A |
| **Study characteristics** | 17 | Cite each included study and present its characteristics. | Pg. 23-25 in the MS; table 1 |
| **Risk of bias in studies** | 18 | Present assessments of risk of bias for each included study. | pg. 7 in the suppl. |
| **Results of individual studies** | 19 | For all outcomes, present, for each study: (a) summary statistics for each group (where appropriate) and (b) an effect estimates and its precision (e.g. confidence/credible interval), ideally using structured tables or plots. | N/A |
| **Results of syntheses** | 20a | For each synthesis, briefly summarise the characteristics and risk of bias among contributing studies. | pg. 7 in the suppl. |
|  | 20b | Present results of all statistical syntheses conducted. If meta-analysis was done, present for each the summary estimate and its precision (e.g. confidence/credible interval) and measures of statistical heterogeneity. If comparing groups, describe the Immediateion of the effect. | Table 2, in the MS; fig. 2-6 in the MS; |
|  | 20c | Present results of all investigations of possible causes of heterogeneity among study results. | N/A |
|  | 20d | Present results of all sensitivity analyses conducted to assess the robustness of the synthesized results. | N/A |
| **DISCUSSION** | | | |
| **Discussion** | 23a | Provide a general interpretation of the results in the context of other evidence. | pg. 13-16 in the MS |
|  | 23b | Discuss any limitations of the evidence included in the review. | pg. 16 in the MS |
|  | 23c | Discuss any limitations of the review processes used. | pg. 16 in the MS |
|  | 23d | Discuss implications of the results for practice, policy, and future research. | pg. 16-17 in the MS |

| **Topic** | **No.** | **Item** | **Location where item is reported** |
| --- | --- | --- | --- |
| **OTHER INFORMATION** | | | |
| **Registration and protocol** | 24a | Provide registration information for the review, including register name and registration number, or state that the review was not registered. | N/A |
|  | 24b | Indicate where the review protocol can be accessed, or state that a protocol was not prepared. | N/A |
|  | 24c | Describe and explain any amendments to information provided at registration or in the protocol. | N/A |
| **Support** | 25 | Describe sources of financial or non-financial support for the review, and the role of the funders or sponsors in the review. | None |
| **Competing interests** | 26 | Declare any competing interests of review authors. | pg. 2 in the MS; |
| **Availability of data, code and other**  **materials** | 27 | Report which of the following are publicly available and where they can be found: template data collection forms; data extracted from included studies; data used for all analyses; analytic code; any other materials used in the review. | Not Available |
| **Availability of data, code and other**  **materials** | 27 | Report which of the following are publicly available and where they can be found: template data collection forms; data extracted from included studies; data used for all analyses; analytic code; any other materials used in the review. | Not Available |

Abbreviations: MS, manuscript; suppl., supplement.

## Supplementary Table S2.PRISMA Abstract Checklist.

| **Topic** | **No.** | **Item** | **Reported** |
| --- | --- | --- | --- |
| **TITLE** | | | |
| **Title** | 1 | Identify the report as a systematic review. | Yes |
| **BACKGROUND** | | | |
| **Objectives** | 2 | Provide an explicit statement of the main objective(s) or question(s) the review addresses. | Yes |
| **METHODS** | | | |
| **Eligibility criteria** | 3 | Specify the inclusion and exclusion criteria for the review. | Yes |
| **Information sources** | 4 | Specify the information sources (e.g. databases, registers) used to identify studies and the date when each was last searched. | Yes |
| **Risk of bias** | 5 | Specify the methods used to assess risk of bias in the included studies. | No |
| **Synthesis of results** | 6 | Specify the methods used to present and synthesize results. | Yes |
| **RESULTS** | | | |
| **Included studies** | 7 | Give the total number of included studies and participants and summarise relevant characteristics of studies. | Yes |
| **Synthesis of results** | 8 | Present results for main outcomes, preferably indicating the number of included studies and participants for each. If meta-analysis was done, report the summary estimate and confidence/credible interval. If comparing groups, indicate the Immediateion of the effect (i.e. which group is favoured). | Yes |
| **DISCUSSION** | | | |
| **Limitations of evidence** | 9 | Provide a brief summary of the limitations of the evidence included in the review (e.g. study risk of bias, inconsistency and imprecision). | No |
| **Interpretation** | 10 | Provide a general interpretation of the results and important implications. | No |
| **OTHER** | | | |
| **Funding** | 11 | Specify the primary source of funding for the review. | No |
| **Registration** | 12 | Provide the register name and registration number. | No |

##

## Supplementary Table S3.Details of the search strategy.

| **Database** | **Search String** |
| --- | --- |
| Pubmed | ("Anxiety"[Title/Abstract] OR "preoperative anxiety" OR "anticipatory anxiety"[Title/Abstract] OR "anxiety disorder"[Title/Abstract] OR "anxiety neurosis" [Title/Abstract] OR anxious*[Title/Abstract] OR panic[Title/Abstract]) AND ("delirium"OR "anesthetic drug"OR "analgesic" OR "pain" OR "recovery time" OR "analgesic*"[Title/Abstract] OR "delirium*"[Title/Abstract] OR "anesthetic"[Title/Abstract] OR "pain"[Title/Abstract] OR "recovery time"[Title/Abstract] OR "anesthetic drug"[Title/Abstract]). |
| Scopus | (Anxiety OR "preoperative anxiety" OR "anticipatory anxiety" OR "anxiety disorder" OR "anxiety neurosis anxiet*" OR anxious* OR panic) AND ("deliriumOR "anesthetic drug"OR "analgesic"" OR pain OR "recovery time" OR analgesic* OR delirium* OR anesthetic OR pain OR "recovery time" OR "anesthetic drug") . |
| Web of Science | (TITLE-ABS(Anxiety) OR "preoperative anxiety" OR TITLE-ABS("anticipatory anxiety") OR TITLE-ABS("anxiety disorder") OR TITLE-ABS("anxiety neurosis anxiet*") OR TITLE-ABS(anxious*) OR TITLE-ABS(panic)) AND ("deliriumOR "anesthetic drug"OR "analgesic"" OR pain OR "recovery time" OR TITLE-ABS(analgesic*) OR TITLE-ABS(delirium*) OR TITLE-ABS(anesthetic) OR TITLE-ABS(pain) OR TITLE-ABS("recovery time") OR TITLE-ABS("anesthetic drug")) . |

##

## Supplementary Table S4. Quality assessment (New-castle Ottowa scale) for Cohort studies.

| **COHORT STUDIES** | **SELECTION** | **COMPARABILITY** | **OUTCOME** | **TOTAL POINTS** |
| --- | --- | --- | --- | --- |
| **Author - Year** |  |  |  |  |
| Ersoy 2024 | 4 | 0 | 3 | 7 |
| Sahin 2023 | 4 | 0 | 3 | 7 |
| Prayunato 2023 | 4 | 0 | 3 | 7 |
| Mou 2023 | 4 | 0 | 3 | 7 |
| Arunalatha 2023 | 4 | 0 | 3 | 7 |
| Kashif 2022 | 4 | 0 | 3 | 7 |
| Fukunaga 2022 | 4 | 0 | 3 | 7 |
| Ali 2022 | 4 | 1 | 3 | 8 |
| Ren 2021 | 4 | 0 | 3 | 7 |
| Ma 2021 | 4 | 0 | 3 | 7 |
| Inal 2021 | 4 | 0 | 3 | 7 |
| Cheng 2021 | 4 | 0 | 3 | 7 |
| Uysal 2020 | 4 | 0 | 3 | 7 |
| Jooma 2020 | 3 | 0 | 3 | 6 |
| Milisen 2020 | 4 | 1 | 3 | 8 |
| Wada 2019 | 4 | 0 | 3 | 7 |
| Masri 2018 | 3 | 0 | 3 | 6 |
| Barreto 2018 | 4 | 0 | 3 | 7 |
| Manjunatha 2017 | 3 | 0 | 3 | 6 |
| Ali 2014 | 4 | 0 | 3 | 7 |
| Van Grootven B 2016 | 4 | 0 | 3 | 6 |
| Gras 2010 | 3 | 0 | 3 | 6 |
| Osborn 2004 | 3 | 0 | 3 | 6 |
| Maranets 1999 | 3 | 0 | 3 | 6 |

##

## Supplementary figures S5. Leave-many-out sensitivity analysis sensitivity analysis was done by removing studies that observed patients undergoing minor surgeries namely: Jooma 2020, Sahin 2023, Inal 2021 and Osborn 2004. Analyses where only one study was left were not included.


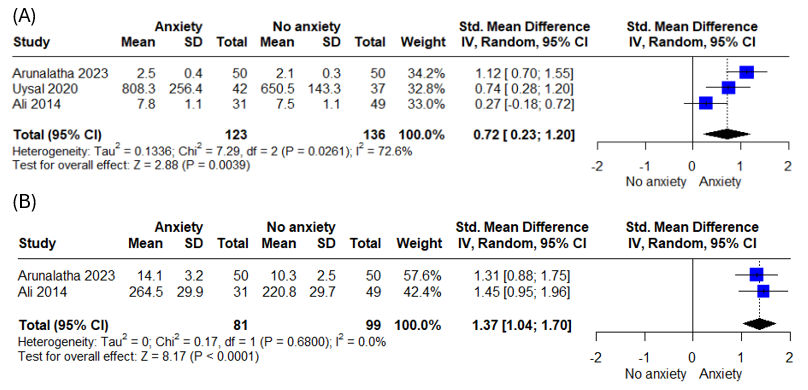


**Figure S5A.** (A) Forest plot of anesthetic drug dose requirements. The x-axis represents the standardized mean difference (SMD) in anesthetic drug dose between anxious and non-anxious patients. (B) Forest plot of analgesic drug dose requirements. The x-axis represents the SMD in analgesic drug dose between anxious and non-anxious patients. For both plots, squares represent individual studies, with size proportional to study weight. Diamond represents the pooled effect size. Horizontal lines represent 95% confidence intervals.


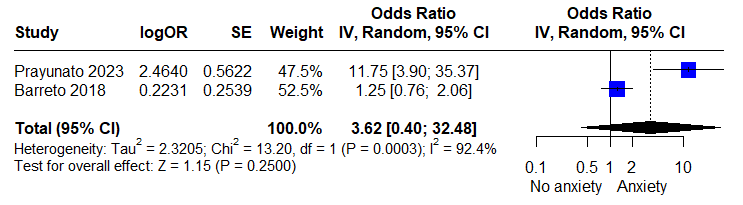


**Figure S5B.** Forest plot of post-operative delirium occurrence in pediatrics. The x-axis represents the odds ratio (OR) of delirium occurrence in anxious versus non-anxious patients. Squares represent individual studies, with size proportional to study weight. Diamond represents the pooled effect size. Horizontal lines represent 95% confidence intervals.


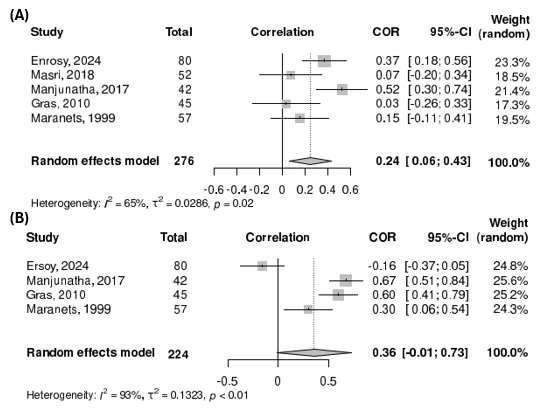


**Figure S5C.** (A) Forest plot and meta-analysis of correlation coefficients testing the relationship between pre-op state anxiety (STAI-S scale) and Propofol consumption (B) Forest plot and meta-analysis of correlation coefficients testing the relationship between pre-op trait anxiety (STAI-T scale) and propofol consumption. For both plots, squares represent individual studies, with size proportional to study weight. Diamond represents the pooled effect size. Horizontal lines represent 95% confidence intervals


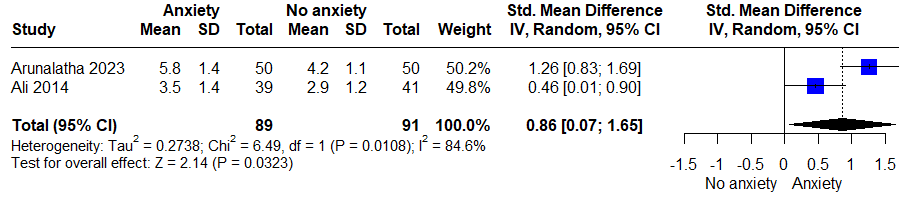


**Figure S5D.** Forest plot of pain 1h post-operatively, squares represent individual studies, with size proportional to study weight. Diamond represents the pooled effect size. Horizontal lines represent 95% confidence intervals
